# Formation, Structure and Magnetic Properties of MFe_2_O_4_@SiO_2_ (M = Co, Mn, Zn, Ni, Cu) Nanocomposites

**DOI:** 10.3390/ma14051139

**Published:** 2021-02-28

**Authors:** Thomas Dippong, Erika Andrea Levei, Oana Cadar

**Affiliations:** 1 Faculty of Science, Technical University of Cluj-Napoca, 76 Victoriei Street, 430122 Baia Mare, Romania; 2 INCDO-INOE 2000, Research Institute for Analytical Instrumentation, 67 Donath Street, 400293 Cluj-Napoca, Romania; erika.levei@icia.ro (E.A.L.); oana.cadar@icia.ro (O.C.)

**Keywords:** ferrite, nanocomposite, thermal behavior, crystallinity, magnetic properties

## Abstract

The formation, structure, and thermal and magnetic properties of MFe_2_O_4_@SiO_2_ (M = Co, Mn, Zn, Ni, Cu) (60% MFe_2_O_4_/40% SiO_2_) nanocomposites produced by a modified sol-gel method, followed by annealing at 300, 600, 900 and 1200 °C, were studied. The thermal analysis and Fourier transform infrared spectroscopy showed the formation of metal-glyoxylates below 210 °C and their decomposition into the corresponding ferrite around 300 °C. The evolution of crystalline phases and variation of crystallite sizes differs from ferrite to ferrite and depends on the annealing temperature. The magnetic measurements revealed the dependence of saturation and remanent magnetization, coercivity, and anisotropy on ferrite type, annealing temperature, and particle size. By annealing the nanocomposites (NCs) at 1200 °C paramagnetic MnFe_2_O_4_, CoFe_2_O_4_, NiFe_2_O_4_ and CuFe_2_O_4_ and antiferromagnetic ZnFe_2_O_4_ are obtained.

## 1. Introduction

The MFe_2_O_4_@SiO_2_ (M = Co, Cu, Mn, Ni, Zn) spinel ferrites possess a cubic structure with tightly packed arrangement of oxygen atoms and metal ions occupying tetrahedral (A) or octahedral (B) sites [1]. Due to their distinctive structure and magnetic, optical, and electrical properties, the transitional divalent metal ferrites are promising functional materials for many applications [1,2,3,4,5,6,7,8,9,10]. Among different coating materials, SiO_2_ is a notable surface modifier due to its exceptional stability, easy conjugation capacity with numerous functional groups, selective and specific coupling ability with biotargets, non-toxicity, and biocompatibility [11]. Mesoporous SiO_2_ is a versatile nanocarrier candidate as it increases the biocompatibility of the incorporated nanoparticles, minimizes their agglomeration, and improves their stability [5]. Polymerized tetraethoxysilane network is often used as surface coating material for Fe oxide nanocrystals, preventing agglomeration and improving chemical stability [12]. The synthesis of SiO_2_ particles with magnetic core by tailoring the silica shell thickness and surface properties was reported by Philipse et al. [11]. At low temperature, the magnetic behavior of SiO_2_-coated ZnFe_2_O_4_ nanoparticles is governed by the core-shell interactions [6]. The saturation (*M_s_*) and remanent (*M_R_*) magnetizations are influenced by the silica coating, whereas the coercivity (*H_c_*) of silica-coated and uncoated CoFe_2_O_4_ are comparable, and that of MnFe_2_O_4_ decreases by coating with silica [12]. The magnetic behavior of coated and uncoated nanoparticles is extensively studied both by experimental measurements and by numerical modeling, but the influence of interparticle interaction on the magnetic properties is not yet fully understood [13]. In the case of SiO_2_-coated CoFe_2_O_4_, the SiO_2_ network shields the nanoparticles and diminishes the surface roughness and spin disorder leading to higher *H_c_* value of CoFe_2_O_4_@SiO_2_ compared to that of CoFe_2_O_4_ [9]. The low saturation magnetization (*M_S_*) of MnFe_2_O_4_@SiO_2_ nanocomposites is attributed to the presence of non-magnetic SiO_2_ matrix, while the *H_c_* value of MnFe_2_O_4_@SiO_2_ nanocomposites is lower than the *H_c_* of unembedded magnetic nanoparticles [5,12]. As the distribution of particle size highly influences the magnetic properties, by tailoring these parameters, different magnetic nanomaterials can be produced [14].

Numerous synthesis methods have been reported for divalent metal (Co, Mn, Zn, Cu, Ni) ferrite nanoparticles, such as sol-gel, coprecipitation, ball milling, autocombustion, reverse micelles, microwave plasma assisted process, sonochemical, hydrothermal, solvothermal, co-precipitation, microemulsion, laser ablation, spray pyrolysis, etc. [1,2,3,4,5,6]. Among these methods, the sol-gel route is a favorable method for ferrite nanocomposite preparation due to its low cost; simplicity; and good control over the structure, physical–chemical, and surface properties. However, nanoparticles of irregular shape may result as a consequence of the large amount of gas evolved during organic solvent decomposition of viscous sol spreading into nanopores by weak capillary forces [2]. The modified sol-gel method consists of the mixing of reactants with tetraethylorthosilicate (TEOS), gelation of the silica network, followed by the thermal-assisted formation of glyoxylate precursors and their decomposition into a simple or mixed oxidic system. In case of ferrites, the modified sol-gel method provides key benefits, such as obtaining pure and homogeneous silica coated nanoparticles, versatility, simplicity and effectiveness, and reduced time and energy, while the main drawbacks are the presence of secondary crystalline phases at high annealing temperatures and amorphous phases at low annealing temperatures.

Cobalt ferrite (CoFe_2_O_4_, CFO), nickel ferrite (NiFe_2_O_4_, NFO), and copper ferrite (CuFe_2_O_4_, CuFO) have inverse spinel structure with 8 M^2+^ (M = Co, Cu, Ni) ions occupying the octahedral sites and 16 Fe^3+^ ions distributed between the tetrahedral and octahedral sites [4,15]. The inverse spinel structure is associated with an assembly of outstanding magnetic properties; good chemical, mechanical, and thermal stability; high electrical resistance; high resistance to corrosion; low eddy current losses; and low production cost [2,3,16]. These properties make them suitable for applications such as sodium-ion batteries, microwave absorbers, magnetic liquids, magnetic refrigeration, magnetic storage, dye removal, enhancement of water oxidation processes, magnetic recording, photocatalysis, ferrofluid technology, medical diagnostics, etc. [8,17,18,19,20]. Depending on the size and shape of particles, NFO exhibits paramagnetic or ferromagnetic behavior, while CuFO exhibits ferromagnetic behavior [18].

Manganese ferrite (MnFe_2_O_4_, MFO) has a partially inverse spinel structure with 20% of Mn^2+^ ions occupying octahedral sites and the other 80% tetrahedral sites [5]. It has attracted considerable attention due its controllable size and shape, high *M_S_* value, paramagnetic character, easy and convenient synthesis, surface tailoring possibility, and good biocompatibility. Moreover, MnFe_2_O_4_ is a non-toxic, non-corrosive, heat-resistant, and environmentally friendly material that is used in the ceramic and paint industry as black pigment [5,21,22]. The embedding of MnFe_2_O_4_ in mesoporous SiO_2_ enhances the nanoparticles stability in water, improves the biocompatibility, and minimizes the agglomeration and degradation [5].

Zinc ferrite (ZnFe_2_O_4_, ZFO) is a normal spinel ferrite, which displays thermal and chemical stability and excellent structural, magnetic, optical, electrical, and dielectric properties [4,6,9,10,23]. Population of tetrahedral (A) sites with non-magnetic Zn^2+^ ions forces the Fe^3+^ ions into octahedral (B) sites, which further leads to weak exchange interactions between Fe^3+^ ions in octahedral sites and results in antiferromagnetic behavior below 9–11 K [11,12,24]. As the octahedral sites are edge-sharing and octahedrally-coordinated with rather short B–B distances, the dominance of the nearest-neighbor interaction is reduced, with the ZnFe_2_O_4_ exhibiting long range order and complex ground states [11,24,25]. Although, the existence of both long- and short-range orders was confirmed, the formation mechanism of short-range order is still under discussion. In the case of nanoparticles, the situation is more complex due to cation distribution inversion, size effects, and non-stoichiometry [26].

This study aims to perform a comparative analysis of structural, morphological, and magnetic properties of nanosized CoFe_2_O_4_, MnFe_2_O_4_, NiFe_2_O_4_, CuFe_2_O_4,_ and ZnFe_2_O_4_, embedded in a SiO_2_ matrix, obtained by sol-gel method followed by thermal treatment at 300, 600, 900, and 1200 °C. Thermal analysis and Fourier transform infrared (FT-IR) spectroscopy data were used to reveal the different formation and decomposition behaviors of metal-glyoxylates and to confirm the ferrite formation. The X-ray diffraction (XRD) analysis revealed the formation of main crystalline phase and allowed the calculation of crystallite size. The study brings valuable information on the changes of structure and magnetic properties of different transitional metal ferrites annealed at different temperatures.

## 2. Materials and Methods

Fe(NO_3_)_3_∙9H_2_O, Co(NO_3_)_2_∙6H_2_O, Mn(NO_3_)_2_∙3H_2_O, Zn(NO_3_)_2_·6H_2_O, Ni(NO_3_)_2_∙6H_2_O, Cu(NO_3_)_2_∙3H_2_O, ethylene glycol (EG), tetraethylorthosilicate (TEOS), ethanol, and 65% HNO_3_ purchased from Merck (Darmstadt, Germany) were used for the synthesis. The purity of all reagents was higher than 98%.

The CFO, MFO, ZFO, NFO, and CuFO (60% MFe_2_O_4_/40% SiO_2_) were synthesized using metal nitrates in 1M/2Fe molar ratio, where M = Co^2+^, Mn^2+^, Zn^2+^, Ni^2+^, or Cu^2+^ by sol-gel method. In all cases, a molar ratio of 1 NO_3_^−^/1 EG/ 0.67 TEOS was used. The resulted sols were kept at room temperature until gelation (8 weeks), ground, dried at 40 °C (5 h) and annealed at 300, 600, 900, and 1200 °C (6 h) in air using a LT9 muffle furnace (Nabertherm, Lilienthal, Germany).

The carboxylate-type precursors formation and decomposition were investigated by thermogravimetry (TG) and differential thermal analysis (DTA) in air, up to 1000 °C, at 10 °C·min^−1^ using alumina standards and Q600 SDT (TA Instruments, New Castle, DE, USA) analyzer. The crystalline phases were investigated by X-ray diffraction using a D8 Advance (Bruker, Karlsruhe, Germany) at ambient temperature with CuKα radiation (λ = 1.54060 Å) and LynxEye detector, operating at 40 kV and 40 mA. The vibration of chemical bonds from the ferrite structure and SiO_2_ matrix were investigated on KBr pellets containing 1% sample using a Spectrum BX II Fourier transform infrared spectrometer (FT-IR, Perkin Elmer, Waltham, MA, USA). The shape and clustering of nanoparticles were studied on samples deposited and dried on carbon coated copper grids using a HD-2700 (Hitachi, Tokyo, Japan) transmission electron microscope (TEM). The magnetic measurements were performed using a 7400 vibrating-sample magnetometer (VSM, LakeShore Cryotronics, Westerville, OH, US). The hysteresis loops were recorded at room temperature in magnetic fields between −2 to 2 T, while the magnetization versus magnetic field measurements were performed to find *M_S_* up to 5 T on samples embedded in epoxy resin. The Co/Fe (CoFe_2_O_4_@SiO_2_), Mn/Fe (MnFe_2_O_4_@SiO_2_), Zn/Fe (ZnFe_2_O_4_@SiO_2_), Ni/Fe (NiFe_2_O_4_@SiO_2_), and Cu/Fe (CuFe_2_O_4_@SiO_2_) molar ratio in the synthesized were confirmed by inductively coupled plasma optical emission spectrometry (ICP-OES) using an Optima 5300DV (Perkin Elmer, Norwalk, CT, USA) after microwave digestion of 50 mg sample with 21 mL aqua regia (7mL HNO_3_ and 21 mL HCl) and dilution with 100 mL ultrapure water.

## 3. Results and Discussion

### 3.1. Thermal Analysis

**Figure 1:**
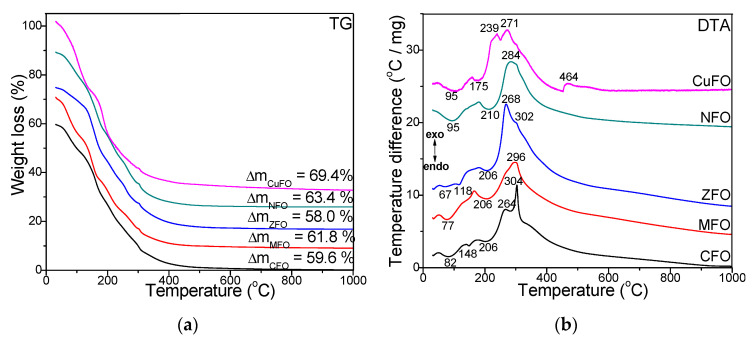
Thermogravimetry (TG) (**a**) and differential thermal analysis (DTA) (**b**) diagrams of cobalt ferrite (CFO), zinc ferrite (ZFO), nickel ferrite (NFO), manganese ferrite (MFO), and copper ferrite (CuFO) samples dried at 40 °C.

Figure 1 shows the TG and DTA curves of CFO, MFO, ZFO, NFO, and CuFO samples dried at 40 °C. The DTA diagrams (Figure 1b) show three processes: (1) loss of physically adsorbed water shown by the endothermic effect at 67–95 °C; (2a) formation, in a single stage, of Cu/Ni/Mn- and Fe-glyoxylates shown by the broad endothermic effect at 175/210/206 °C, due to the overlapping of effects attributed to M-glyoxylates with Fe-glyoxylate for MFO, NFO, and CuFO nanocomposites (NCs) or (2b) formation, in two stages, of Fe-glyoxylate indicated by the endothermic effect at 118 °C (ZFO), 148 °C (CFO) and M-glyoxylates at 206 °C (ZFO and CFO); (3) decomposition of glyoxylate precursors to NFO and MFO indicated by a broad exothermic effect at 284 and 296 °C, respectively. The formation of CFO occurs in two well-delimited stages by the decomposition of Co-glyoxylate to CoO (exothermic effect at 264 °C) and Fe-glyoxylate to Fe_2_O_3_ (exothermic effect at 304 °C), which finally forms CoFe_2_O_4_. A similar trend was observed for ZFO and CuFO, suggesting the decomposition of Zn-glyoxylate to ZnO (at 268 °C) and Cu-glyoxylate to CuO (at 239 °C) and Fe glyoxylate to Fe_2_O_3_ (at 302 and 271 °C, respectively) [2,27,28,29]. In case of CuFO, a fourth process indicated by the exothermic effect at 464 °C is assigned to phase transformations in ferrite and SiO_2_ matrix [2]. According to TG diagrams (Figure 1a), the lowest total mass loss was remarked in the case of ZCF (58.0%), while the highest total mass loss was found in the case of CuFO (69.4%).

### 3.2. FT-IR Analysis

**Figure 2:**
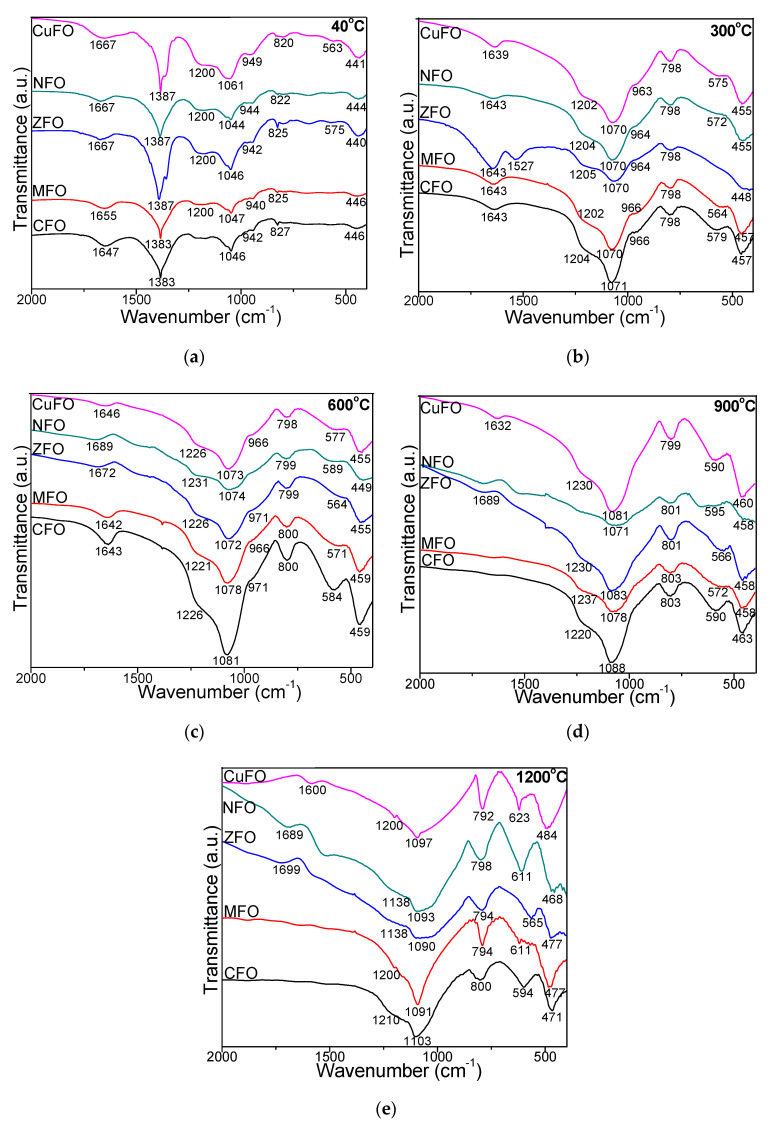
Fourier transform infrared (FT-IR) spectra of CFO, MFO, ZFO, NFO, CuFO samples thermally treated at 40 °C (**a**), 300 °C (**b**), 600 °C (**c**), 900 °C (**d**), and 1200 °C (**e**).

The FT-IR spectra of samples dried at 40 °C displayed an intense band at 1387 cm^−1^ attributed to the vibration of N–O bonds of nitrate groups (Figure 2). This band disappeared for samples annealed at higher temperatures, confirming the conversion of nitrates [2]. In all cases, the formation of SiO_2_ matrix was indicated by its characteristic bands. The bands at 792–827 cm^−1^ were attributed to symmetric stretching/bending vibrations of Si–O chains in SiO_4_ tetrahedron, the bands at 1046–1103 cm^−1^ to the stretching/ bending vibrations of Si–O–Si bonds, while the shoulder at 1200–1237 cm^−1^ to the vibration of Si–O bonds in SiO_2_. In the cases of CuFO, CFO, and MFO, the bands at 1046–1103 cm^−1^ increased with the increase of annealing temperature, while the shoulder attributed to the vibration of Si–OH bonds (942–971 cm^−1^) disappeared at high temperatures (900 and 1200 °C). Dissimilarly, for NFO and ZFO annealed at 1200 °C this band shifted to 1138 cm^−1^. In all cases, a possible explanation could be the different bond strength of metal and oxygen atoms that further leads to distinct bond length in SiO_2_ matrix, resulting in minor variations of the peak position for each ferrite. Ferrites can be called continuously bonded crystals with the atoms bonded to all nearest neighbors by equal forces [2,30,31]. Additionally, the Si–O–Si bond length variations under the influence of the neighboring atom could produce the peak wavenumber shift [2,31]. The band at 1643–1699 cm^−1^ corresponding to the stretching mode vibration of surface-adsorbed H–O–H molecules disappeared in the case of CFO and MFO annealed at 900 and 1200 °C [1,2,7]. The band at 563–623 cm^−1^ is specific to tetrahedral stretching vibration of Co–O, Zn–O, Mn–O, Zn–O, Cu–O bonds. After annealing at 1200 °C, the vibration of Mn–O, Ni–O and Cu–O bands shifted towards higher wavenumbers. The bands at 440–488 cm^−1^ are attributable to the octahedral stretching vibration of Fe–O bond [8,19]. This slight shift towards low wavenumbers with the increase of annealing temperature suggests changes in crystallite size and M–O bond length in ferrite as a consequence of changes in the spinel structure [23].

### 3.3. Chemical Analysis

The M/Fe molar ratio calculated based on Co, Fe, Mn, Zn, Ni, and Cu concentrations measured by ICP-OES confirmed the theoretical elemental composition of the obtained NCs (Table 1). The best fit of experimental and theoretical data was remarked for samples annealed at 1200 °C.

### 3.4. XRD Analysis

**Figure 3:**
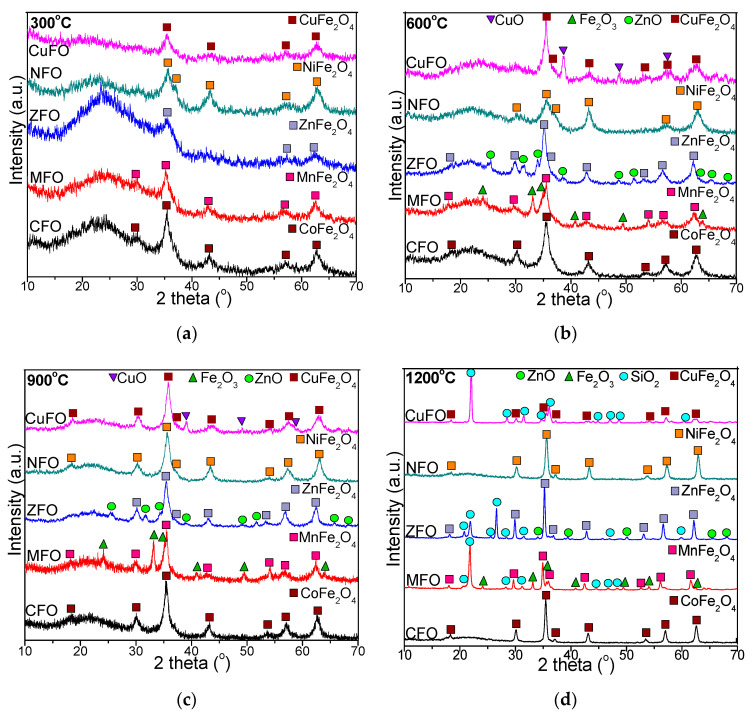
X-ray diffraction (XRD) patterns of CFO, MFO, ZFO, NFO, CuFO NCs annealed at 300 °C (**a**), 600 °C (**b**), 900 °C (**c**), and 1200 °C (**d**).

The XRD patterns show the crystallographic properties of transition metal ferrite nanostructures, providing significant information about the structure, crystal orientation, the average crystallite size, etc. [6]. The evolution of crystalline phases in CFO, MFO, ZFO, NFO, and CuFO is presented in Figure 3. At high annealing temperatures, the presence of well-defined and narrow peaks indicated well crystallized particles. By low temperature annealing only low crystalline phases were obtained [1]. The XRD pattern of CFO exhibited a broad diffraction halo in the range 15°–30° corresponding to the amorphous SiO_2_ matrix and cubic spinel CoFe_2_O_4_ crystalline single phase (JCPDS card no. 02-1045, [32])) indexed to (111), (220), (311), (222), (400), (422), (511), and (440) planes, belonging to Fd3m space group [2,3,33]; this confirms the good homogeneity and crystallization of the prepared compound [17]. The single CoFe_2_O_4_ phase started to form at 300 °C due to the strong reducing atmosphere (CO) generated during decomposition, when amorphous, very reactive CoO and γ-Fe_2_O_3_ were formed [34,35,36,37,38]. In the case of MFO, a less intense amorphous halo and low crystalline MnFe_2_O_4_ single phase (JCPDS card no. 89-3434, [32]) indexed to (220), (311), (400), (422), (511), and (440) planes of spinel cubic MnFe_2_O_4_ were observed [5,22]. Additionally, in the case of ZFO, a decrease of the intensity of the diffraction halo at 15°–30° was observed beside the barely visible lines of single ZnFe_2_O_4_ phase (JCPDS card no. 16-6205 [28]) indexed to (220), (311), (222), (400), (422), (511), (440), and (533) planes. In the case of NFO, the Bragg reflection peaks were indexed to low-crystallized single NiFe_2_O_4_ phase (JCPDS card no 89-4927) [28]), face centered cubic, and Fd3m space group, displaying typical reflections of (220), (311), (222), (400), (422), (511), and (440) planes [4,18]. In the case of CuFO, the XRD peaks exhibited broadened reflections of (111), (220), (311), (222), (400), (511), (440), and (533) planes corresponding to the cubic spinel single phase structure of CuFe_2_O_4_ (JCPDS card no. 25-0283 [32]) [4,8]. A sharp diffraction peak overlaid on a broad base (such as 311 peak) indicated the presence of both well- and low crystalline phases due to the overlapping between the narrow, high-intensity and broad, low-intensity peaks [5]. In the case of samples annealed at 600 °C and 900 °C, a significant decrease in the intensity of the diffraction halo characteristic to the amorphous SiO_2_ matrix compared to 300 °C was observed. In the cases of CFO and NFO, the formation of single, well-crystallized CoFe_2_O_4_ and NiFe_2_O_4_ phases was observed. Dissimilarly, in the cases of MFO, ZFO and CuFO, beside the main MnFe_2_O_4_, ZnFe_2_O_4_ and CuFe_2_O_4_ phases, a secondary phase was remarked, as follows: Fe_2_O_3_ (JCPDS card no. 87-1164 [32]), ZnO (JCPDS card no 89-1397 [32]) and a prominent monoclinic CuO phase (JCPDS card no 89-5895) [8,32]. The presence of CuO could be a consequence of the larger complexation constant of Fe^3+^ compared to that of Cu^2+^, leading to more stable Fe^3+^ complexes than the corresponding Cu^2+^ complexes [8].

At 1200 °C, the NCs were well-crystallized, indicating the formation of single CoFe_2_O_4_ and NiFe_2_O_4_ phases for CFO and NFO, respectively. The peaks attributed to CoFe_2_O_4_ were intense and sharp, indicating their high crystallinity [1]. In the case of MFO, beside the main MnFe_2_O_4_ phase, two secondary phases of Fe_2_O_3_ and SiO_2_ (JCPDS card no. 89-3434 [2,32]) were observed. A possible explanation of this distinct formation of a mixed secondary phase of Fe_2_O_3_ and SiO_2_ could be the instability of Mn^2+^ ions. The SiO_2_ matrix produces steric repulsion between nanoparticles, preventing uncontrolled aggregation [27]. The oxidation-reduction reactions also depend on the oxygen partial pressure and the presence of air during the annealing process [17]. In the cases of ZFO and CuFO, beside the main ZnFe_2_O_4_ and CuFe_2_O_4_ phases, a secondary SiO_2_ (α-cristobalite) phase was also present. Crystalline phases resulting following the interaction of ferrite with the SiO_2_ matrix were not noticed. The width of the most intense peak (311) progressively decreased, and the intensity of diffraction lines increased with the increase of annealing temperature and appearance of SiO_2_ crystalline phase. At high annealing temperatures, the intensity of diffraction peaks increased due to the higher crystallinity and inactive surface layer of the crystals, whereas the coalescence processes strengthened, facilitating the increase of grain size [1,2,3,4,5,6]. In this regard, at 1200 °C, a substantial agglomeration occurred without recrystallization, favoring the formation of single crystals instead of polycrystals [1,3,6]. Consequently, the annealing temperature plays a crucial role in governing the crystallinity and crystallite size [16].

The crystallite size is an important parameter to tailor the magnetic and optical properties of ferrites [4,5,6]. The average crystallite size (D_CS_) was calculated using the most intense diffraction peak of (311) crystalline plane by Debye–Scherrer formula using Equation (1) [1,4,6,7,8,15,16,17,20,23,33,34,35,36,37,38]. The lattice parameter (a) calculated according to Equation (2) [6,7,16] is presented in Table 2.

(1) $$D_{\mathrm{CS}}=\frac{0.9\;\lambda }{\beta \;\cos\;\theta }$$

where D_CS_ is average crystallite diameter, β is the broadening of full width at half the maximum intensity (FWHM), θ is the Bragg angle, and λ is the X-ray wavelength.

(2) $$a=\frac{\lambda }{2\sin\theta }\sqrt{h^{2}+k^{2}+l^{2}}$$

where h, k, and l are the Miller indices, λ is the wavelength of the X rays, and θ is the diffraction angle corresponding to the (h k l) plane.

In all cases, the lattice parameter increased with increasing annealing temperature. The differences between the lattice parameter of ferrites were attributed to the different ionic radii of Fe^3+^ (tetra: 0.49; octa: 0.64 Å), Zn^2+^ (tetra: 0.60; octa: 0.74 Å), Cu^2+^ (tetra: 0.57; octa: 0.73 Å), Ni^2+^ (tetra: 0.54; octa: 0.78 Å), Mn^2+^ (tetra: 0.58; octa: 0.69 Å), and Co^2+^ (tetra: 0.58; octa: 0.74Å) [29,39,40]. The lattice parameter increased as the particle size increases, probably due to the decrease of surface tension caused by the size effect [16,20].

### 3.5. Transmission Electron Microscopy

**Figure 4:**
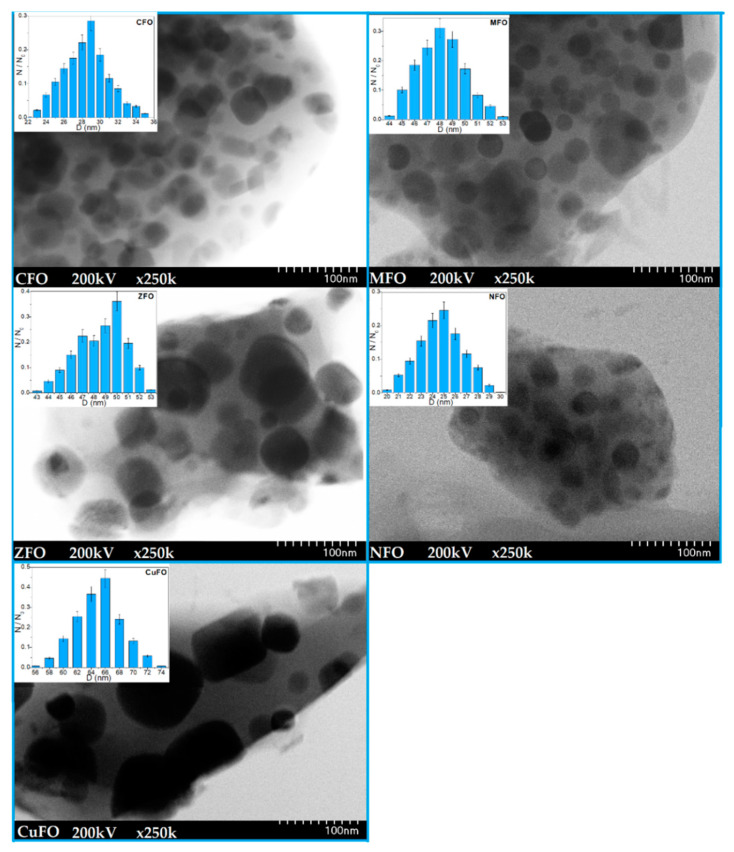
TEM images and particle size distribution for CFO, MFO, ZFO, NFO, and CuFO NCs annealed at 1200 °C.

The TEM images and particle size distribution of studied ferrites are shown in Figure 4.

At 1200 °C, the spherical particles formed irregular spongy aggregates with a large number of pores. The particles were randomly distributed and embedded in the silica matrix. The particle agglomeration was characteristic of chemically prepared NCs and was probably due to the assembling tendency of very small particles [29]. When the nucleation rate was higher than the growth rate, small and homogenously distributed particles were obtained. The histograms show a unimodal particle size distribution for all studied ferrites. The average particle size increased in the order of NiFe_2_O_4_@SiO_2_ (25 nm) > CoFe_2_O_4_@SiO_2_ (29 nm) > MnFe_2_O_4_@SiO_2_ (48 nm) > ZnFe_2_O_4_@SiO_2_ (50 nm) > CuFe_2_O_4_@SiO_2_ (66 nm), The particle sizes estimated by XRD and TEM were comparable, the low differences appearing probably due to the influence of amorphous SiO_2_ and large-size nanoparticles on the diffraction pattern.

### 3.6. Magnetic Behavior

**Figure 5:**
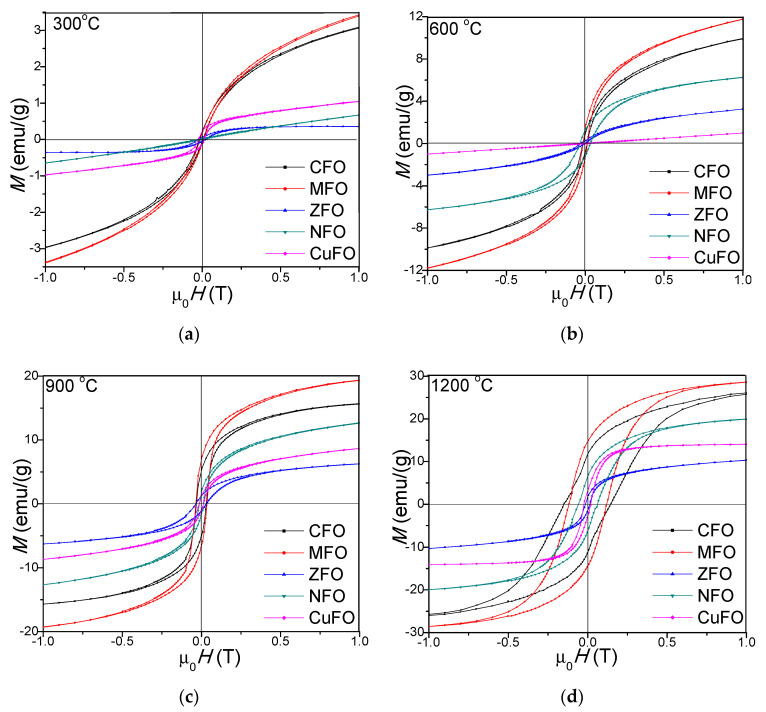
Magnetic hysteresis loops of CFO, MFO, ZFO, NFO, and CuFO NCs annealed at 300 (**a**), 600 (**b**), 900 (**c**), and 1200 °C (**d**).

The magnetic hysteresis loops of CFO, MFO, ZFO, NFO, and CuFO, annealed at 300, 600, 900, and 1200 °C, are presented in Figure 5. The hysteresis loops show that the samples became magnetically softer with increasing temperature [41]. The hysteresis loops had an S-shape at low magnetic fields and linear dependence at high fields, indicating the presence of small magnetic particles [42]. For particles smaller than the critical diameter, the spin-reversal energy was much lower than the thermal energy. In the absence of any magnetic field, the random orientation of the magnetic moments resulted in a zero average magnetic moment [29]. The low saturation magnetization (*M_S_*) values of samples annealed at 300 (0.7–3.6 emu/g), 600 (1.3–11.7 emu/g), and 900 °C (6.1–19.4 emu/g), compared to those annealed at 1200 °C (10.8–28.5 emu/g), could be explained by the low crystallinity, occurrence of vacancies, interatomic spacing, low coordination number, and surface spin disorder [12,29,43]. The MFO, NFO, CFO, and CuFO had paramagnetic behavior between 900 and 1200 °C, and ferromagnetic behavior at lower temperatures, whereas the ZFO was antiferromagnetic at all annealing temperatures. The paramagnetic behavior was determined by the increase of magnetic moment orientation disorder in various sites when the surface/volume ratio increased [38,44]. This behavior resulted from the uncompensated spins of antiferromagnetic clusters, which created giant effective spins that interacted with the applied magnetic field. Since the antiferromagnetic interactions were present both in intra- and inter-cluster spins, the global magnetic behaviors of these samples were found to be different from that of conventional superparamagnetic systems [36]. Also, the presence of stable Mn–O bonds in the crystal system of MnFe_2_O_4_ led to a stable magnetic phase [22].

The low values of *M_S_* for ZFO (0.7–10.8 emu/g) were determined by the lattice defects, particle size effects, core-shell interactions, spin canting, disordered distribution of cations, A–B super exchange interaction, and random spin orientation on the surface of nanoparticles. The magnetic transformation with the increasing annealing temperature in the case of ZFO may be the consequence of the Fe^3+^ and Zn^2+^ ions redistribution in the spinel structure. The different *M_S_* value for ZFO reported in the literature indicates that *M_S_* value strongly depends on different factors, such as the synthesis route, precursors type, thermal treatments, etc. [23,38]. The *M_S_* value of NFO was lower than that of MFO and CFO_4_, but two times higher than that of ZFO, probably due to the increasing surface effects with decreasing particles size [18]. The magnetic performance was greatly influenced by the particle size, due to the formation of a magnetic domain in which all the magnetons are aligned in one direction by exchange force [16]. The *M_s_* increased with increasing crystallite size. The size and shape of nanoparticles changed with increasing annealing temperature, leading to surface effects, spin canting-induced surface disorder, pinning of the magnetic moment, and cation inversion in the spinel ferrite nanostructures. In all cases, the *M_s_* and remanent magnetization (*M_R_*) increased with the increasing annealing temperature, the highest values being found for MFO (*M_s_* = 28.5 emu/g, *M_R_* = 14.83 emu/g) and the lowest for ZFO (*M_s_* = 10.8 emu/g, *M_R_* = 1.67 emu/g) (Table 2). In accordance with Asghar [5] the very low and negligible values of M_R_ suggest a superparamagnetic behavior of the NCs. The *Ms* and *M_R_* increased with the increase of annealing temperature [45]. The low remanent magnetization in the cases of ZFO, NFO, and CuFO could be explained by the low content of magnetic phases present in the nanocomposite [12]. The crystallite size and M_s_ increased with the increase of the annealing temperature as follows: CuFe_2_O_4_ (9.2–60.1 nm, 1.1–14.5 emu/g), NiFe_2_O_4_ (2.8–23.4 nm, 0.7–20.1 emu/g), CoFe_2_O_4_ (7.2–27.8 nm, 3.5–26.3 emu/g), MnFe_2_O_4_ (6.4–45.2 nm, 3.6–28.5 emu/g), and ZnFe_2_O_4_ (4.2–49.5 nm, 0.7–10.8 emu/g). The surface dipole interactions, high surface energy and tension, together with the cation charge distribution within the nanocrystallite induced the reduction of the lattice, which further caused the decrease of the lattice parameter, and blocked the grain growth [1,2,3,4,5,6].

For nano-size particles with multi-axial orientation anisotropy, (K) was calculated from *H_c_* and *M_s_* using Equation (3) [36].

(3) $$K=\frac{M_{s}\cdot H_{c}}{2}$$

The coercivity (*H_c_*) increased with increasing annealing temperature for CFO, MFO, and NFO, indicating a higher degree of disorder in the magnetic moment arrangement at higher annealing temperatures, as a result of higher spin disorder, especially at the surface layer, since *H_c_* was significantly depreciated in smaller size particle where the spin disorder is increased [23]. When the grain size attained the single domain state, the particles exhibited a paramagnetic behavior and the surface effects became dominant over magnetization [23]. The *H_c_* of the spinel ferrite nanoparticles was governed by the magneto-crystalline anisotropy, strain, inter-particle interaction, particle size, and morphology [34]. The *H_c_* also increased with the enhancement of surface potential barrier caused by the crystalline lattice defects in the surface layers. However, the dependence of *H_c_* on the particle size, internal strain, magnetic domain structure, shape, and magnetocrystalline *K* of the particles was not completely elucidated. The low *H_c_* at low annealing temperature in all samples suggested an enhanced coalescence of the crystallites that resulted in stronger magnetic coupling and higher magnetization [42,45]. The *H_c_* and magnetocrystalline *K* of CFO was determined by the Co^2+^ ions located in octahedral sites that further induced frozen orbital angular momentum and strong spin-orbital coupling [16]. The anisotropy constant (*K*) increased with the increase of annealing temperature. Generally, the crystallographic orientations, the presence of defects or inhomogeneities caused the magnetic *K* of samples [46]. This behavior can be related to the influence of cationic stoichiometry and their distribution between the A and B sites, the decrease of magnetocrystalline anisotropy, the occupancy of magnetic cations sites, and the increase of random canting of the surface spins [36]. The modification of surface spin coordination created an important change in magnetic ordering due to the superexchange interaction mediated by oxygen ions and to the broken bonds when oxygen ions were missing from the surface [36,37]. The energy of a magnetic particle in an external field is proportional to the number of magnetic molecules in a single magnetic domain, and in consequence, to its size [36]. The magnetic behavior of the MFO and CFO nanocomposites at 1200 °C is characteristic for hard magnet type ferrites having large hysteresis cycles and high anisotropy [37].

Generally, the magnetic properties of spinel ferrites depend upon the composition, particle size, structure, and cation distribution between octahedral and tetrahedral sites [47]. The increase in *Ms* along the magnetic CoFe_2_O_4_ nanoparticles, with annealing temperature as a consequence of the increase of crystallinity degree and particle size, was also remarked in our previous studies [48]. The *Ms* increased with the increase of the degree of crystallinity due to the surface effect determined by the disorder of surface spins [49]. The increase of *Ms* with the Co ferrite content and annealing temperature was also reported by Salunkhe et al. [44]. For CoFe_2_O_4_, Varna et al. [50] also stated the increase of *Ms* with the annealing temperature and particle size and the decrease of *M_R_* with the increase of particle size. Conversely, in our study, the increase of *M_R_* with the particle size was observed not only for CFO but also for the other studied ferrites. The high *Ms* and low *H_c_* of NiFe_2_O_4_ was attributed to high crystallinity and uniform morphology by Majid et al. [20]. The *H_c_* variation with particle size could be the consequence of the domain structure, critical diameter, and anisotropy of the crystals [51]. The decrease of *H_c_* value could be associated with larger magneto crystalline anisotropy, while the increase of *H_c_* could be associated with the scattering in the anisotropy field directions and with inhomogeneous broadening [52]. The magnetic properties of CuFe_2_O_4_ were also reported to be strongly dependent on particle size, shape, and purity [53]. At temperatures below the Neel temperature, ZnFe_2_O_4_ is antiferromagnetic but converts into diamagnetic, superparamagnetic, or ferromagnetic material when the size of ZnFe_2_O_4_ reaches nanometer level [48,54]. The superparamagnetic behavior of the nanocrystalline ZnFe_2_O_4_ is determined by the increase of the magnetic moment orientation disorder when the ratio surface/volume increases [38].

## 4. Conclusions

In this work, we performed a comparative study of structural, morphological, and magnetic properties of MFe_2_O_4_@SiO_2_ (M = Co, Mn, Zn, Ni, Cu) obtained by a modified sol-gel method, followed by thermal treatment in the range of 300–1200 °C. Additionally, the relationship between crystallite size, lattice parameter, saturation, and remanent magnetizations and magnetic anisotropy was investigated. Thermal analysis indicated interesting, different formation and decomposition behaviors of metal-glyoxylates and, along with Fourier transform infrared (FT-IR) spectroscopy, confirmed the ferrite formation. Also, the study brought valuable information on the changes of structural and magnetic properties of ferrites annealed at different temperatures. Compared to the classical sol-gel synthesis approach, the modified sol-gel method offers important benefits such as reduced time, versatility, and simplicity to obtain pure and homogeneous nanoparticles; though, in the cases of CuFe_2_O_4_, ZnFe_2_O_4_, and MnFe_2_O_4_, secondary crystalline phases at high annealing temperatures and amorphous phases at low annealing temperatures were also noted. The thermal analysis showed the formation of glyoxylate precursors up to 210 °C and their decomposition into ferrites around 300 °C. The Mn and Ni ferrites were obtained by decomposition of the glyoxylate precursors in a single stage, while Co, Cu, and Zn ferrites by the decomposition of glyoxylates in two stages. XRD indicated the formation of single crystalline phases in the cases of Co and Ni ferrites at all annealing temperatures, of poorly crystallized Zn and Cu ferrites at low temperatures, and of crystallized Zn and Cu ferrites accompanied by crystalline ZnO and CuO at 600 and 900 °C and by crystalline SiO_2_ at 1200 °C. In the case of Mn ferrite, the crystalline phase was unpurified by crystalline Fe_2_O_3_ at 600–900 °C and by SiO_2_ at 1200 °C. The crystallite sizes increased with increasing annealing temperature ranging between 9.2–60.1 nm (CuFe_2_O_4_), 4.2–49.5 nm (ZnFe_2_O_4_), 6.4–45.2 nm (MnFe_2_O_4_), 7.2–27.8 nm (CoFe_2_O_4_), and 2.8–23.4 nm (NiFe_2_O_4_). After sample annealing at 1200 °C, the average crystallite size estimated both from XRD and TEM data was comparable. The average particle size increased in the order of: NiFe_2_O_4_@SiO_2_ > CoFe_2_O_4_@SiO_2_ > MnFe_2_O_4_@SiO_2_ > ZnFe_2_O_4_@SiO_2_ > CuFe_2_O_4_@SiO_2_. The *M_S_* and *M_R_* increased with increasing annealing temperature, the highest values being measured for MnFe_2_O_4_ and the lowest values for ZnFe_2_O_4_ in spite of having the largest crystallite sizes. Mn ferrites, Ni ferrites, Cu ferrites, and Co ferrites had paramagnetic behavior above 900 °C and ferromagnetic behavior at lower temperatures, whereas Zn ferrite was antiferromagnetic at all temperatures. The *H_C_* increased with increasing annealing temperature in the cases of CoFe_2_O_4_, MnFe_2_O_4_ and NiFe_2_O_4_, while the magnetic *K* increased with temperature in the case of all ferrites.

## Figures and Tables

**Table 1:** M/Fe molar ratio in the MFe_2_O_4_@SiO_2_ (M = Co, Cu, Mn, Ni, Zn) annealed at 300, 600, 900, and 1200 °C.

Temperature (°C)	Co/Fe	Mn/Fe	Zn/Fe	Ni/Fe	Cu/Fe
300	0.99/2.01	0.97/2.03	0.99/2.01	0.97/2.03	0.96/2.04
600	0.98/2.02	0.97/2.03	0.99/2.01	0.98/2.03	0.97/2.03
900	0.99/2.01	0.98/2.02	1.00/2.00	0.99/2.01	0.98/2.02
1200	1.00/2.00	0.99/2.01	1.00/2.00	0.99/2.01	1.00/2.00

**Table 2:** Average particle size (D*_PS_*), average crystallites size (D_CS_), lattice parameter (a), saturation magnetization (*M_s_*), remanent magnetization (*M_R_*), coercivity (*H_c_*), and magnetic anisotropy constant (K) of CFO, MFO, ZFO, NFO, and CuFO NCs annealed at 300, 600, 900, and 1200 °C.

NC	Temperature (°C)	CFO	MFO	ZFO	NFO	CuFO
D*_PS_* (nm)	1200	29	48	50	25	65
D_CS_ (nm)	300 600 900 1200	7 11 18 28	6 15 33 45	4 11 21 49	3 8 10 23	9 20 38 60
a (Å)	300 600 900 1200	8.223 8.271 8.367 8.438	8.331 8.391 8.434 8.484	8.324 8.365 8.380 8.427	8.319 8.356 8.363 8.382	8.123 8.174 8.214 8.246
*M_s_* (emu/g)	300 600 900 1200	3.5 9.8 15.6 26.3	3.6 11.7 19.4 28.5	0.7 3.3 6.1 10.8	0.7 6.3 12.7 20.1	1.1 1.3 8.6 14.5
*M_R_* (emu/g)	300 600 900 1200	0.25 0.97 5.10 11.96	0.26 1.39 6.66 14.83	0.07 0.31 1.23 1.67	0.03 1.19 1.36 6.82	0.23 0.03 0.92 3.03
*H_c_* (T)	300 600 900 1200	0.015 0.016 0.031 0.144	0.017 0.018 0.035 0.119	0.013 0.003 0.025 0.015	0.016 0.024 0.026 0.061	0.009 0.032 0.019 0.018
*K·10^3^* (erg/cm^3^)	300 600 900 1200	0.033 0.098 0.304 2.378	0.038 0.132 0.426 3.392	0.005 0.006 0.096 0.102	0.007 0.094 0.207 0.770	0.006 0.028 0.103 0.163

## Data Availability

The data presented in this study are available on request from the corresponding author.
